# Load bearing capacity of arch structure in unconsolidated layers

**DOI:** 10.1038/s41598-023-31158-x

**Published:** 2023-03-14

**Authors:** Feng Wang, Weihao Zhu, Zeqi Jie, Lang Lu, Zetao Chen

**Affiliations:** 1 College of Energy and Mining Engineering, Shandong University of Science and Technology, Qingdao, 266590 Shandong China; 2 State Key Laboratory of Mining Disaster Prevention and Control Co-Founded By Shandong Province and the Ministry of Science and Technology, Shandong University of Science and Technology, Qingdao, 266590 Shandong China

**Keywords:** Civil engineering, Coal

## Abstract

Coal mining inevitably results in the movement of overlying strata, with the upward formation of the strata leading to surface subsidence, causing irreversible impact on the buildings, land, and ecological environment. The movement and deformation of the strata are controlled by the bearing structure in the overlying strata, whose failure results in the deformation and breakage of the overlying strata simultaneously. While studies have been conducted on the arch structure in unconsolidated layers (ASUL), its bearing performance has not been addressed. Therefore, this study develops a bearing mechanics model based on the morphological characteristics of the ASUL. The analytical expressions of the axial force, bending moment, and shear force of the cross-sectional area were determined using theoretical derivations. The model analysed the internal forces and showed the influence laws of the overlying load, horizontal pressure coefficient, and rise-to-span ratio of the ASUL. The failure criterion of the bearing was also further determined. The results indicated that with overlying and horizontal loads, the axial force and bending moment are symmetrically distributed, whereas the shear force is asymmetrically distributed. In addition, the axial force gradually increases from the dome to the base of the ASUL. Compared to the axial force and bending moment, the shear force has a lower impact on the stability of the ASUL. Most of the axial force and overlying load is received through the axial compression of the cross-section to maintain stability and play a bearing role on the overlying unconsolidated layers. As the overlying load, horizontal pressure coefficient, and rise-to-span ratio increase, the axial force, bending moment, and shearing force also increase gradually. This effect is more apparent at the dome, spandrel, and base of the ASUL. The stability of the dome and spandrel is key to the overall structural stability. Therefore, the failure criterion for the ASUL was determined based on the compression failure at the dome and spandrel. During the mining process of the working face, the ASUL served as load-bearing control for the overlying unconsolidated layers. Further, increasing width of the working face damages and shifts the base of the ASUL, resulting in compression failure at the dome and spandrel, further inducing dome lift and causing overall failure of the ASUL. Considering the aforementioned factors, a control method that reinforces the surface subsidence of the ASUL by 'one-time, upward, staged, and multiple-ground-drilling' compaction grouting has been proposed. During the mining process of the working face, the arch bead-like structure, combined with the ASUL, serves as the load-bearing control on the overlying strata and ground surface, reducing ASUL deformation in the unconsolidated layers, overlying strata, and ground surface. This process enables the controlling of ground subsidence of coal mining in thick unconsolidated layers.

## Introduction

**Figure 1:**
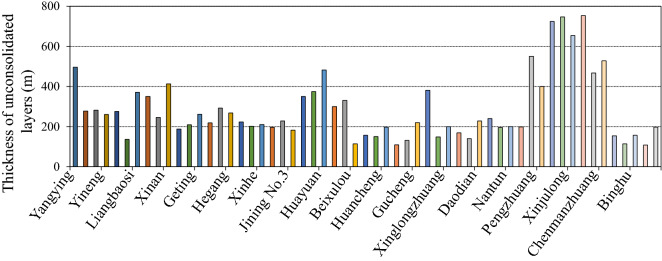
Thickness of unconsolidated layers in coal mines in western Shandong Province coal base.

The increasing number of coal mining activities across the world has induced irreversible negative impact on buildings, land, and ecological environment. The primary factor behind the negative impact is the movement of overlying strata and the upward development of rock strata. Expansive mining regions of thick unconsolidated layers are present in parts of Eastern and Northern China. A total of 57 coal mines in the Shandong Luxi Coal Base, including Liangbaosi, Huayuan, Zhangji, and Xinjulong, have maximum and minimum unconsolidated layer thicknesses of 753 m and 95 m, respectively, with an average thickness of 286 m, as shown in Fig. 1.

The overlying strata movement pattern in the mining areas with thick unconsolidated layers are characterised by sensitivity, severe sinking, subsidence coefficient, and subsidence rate^1–4^. Further, their movement is controlled by the bearing structure. Unlike masonry structures^5^ or other key layer structures^6^ proposed for rock formations based on the beam or slab mechanics models, the mechanical properties of the unconsolidated layers are most similar to those of a random medium^7^. The load-bearing structure for the unconsolidated layers is proposed based on the mechanics of granular media. The unconsolidated layers are the Quaternary and Neoproterozoic strata, primarily comprised of loose sediments that have not yet consolidated and hardened into rocks, whose basic mechanical properties are lower than those of rock strata^8^. The ability of the proposed ASUL to maintain its stability and serve as a stable bearing for the overlying strata and ground surface, based on the mechanics of granular media, must be determined through theoretical and applied research.

Recently, several detailed studies on ASUL have been published. Determined an equation for the morphological characteristics of the ASUL through theoretical analysis and evaluated the impact of its mechanism on the movement and deformation characteristics of the overlying strata as well as its rupture failure^7,9–11^. Developed a mechanical model of the arch structure and derived the relationship between the span and height of the arch structure^12^. Proposed a binary structural model of a beam arch for overlying strata rupture and studied and demonstrated the spatial evolution characteristics of arch structure failure through a discrete element numerical simulation study^13^. Proposed a 'double-arch bridge' model for the roof of a shallowly buried proximity coal seam group and analysed its stability^14^. Developed a mechanical model of the arch structure and obtained its maximum compression deformation under load^15^. Created a mathematical model of the arch beam structure by analysing the damage characteristics of thick sandy soil layers for the surface overload characteristics of the Shendong mining region. The bearing stress components of the and the rupture criterion of the thick sandy soil layers based on the structural mechanics model of the arch beam were determined^16,17^. Developed an analytical model of the stress arch shells for high-dip coal seam mining and obtained the criterion for determining stress arch shell stability. The failure of the stress arch shell was further divided into the 'shell base–shell dome (shell spandrel)' failure mode and 'shell dome (shell spandrel)—shell base' failure modes^18,19^. Proposed a mechanical model of the 'layer-double arch' bearing structure to provide a basis for controlling the surrounding rock in deep mining roadways by determining the characteristics of the bearing stress distribution and analytical expression for the maximum bearing strength of the structure^20^. Proposed an 'arch and shell' equilibrium macrostructure theory for the hard roof rock strata in the Datong mining region. The 'arch and shell' equilibrium macrostructure equation was established, and its mechanical equilibrium condition was determined^21^. These studies have contributed to ASUL stability its application in ground control. A study of the bearing performance of this ASUL will further clarify the characteristics and laws of internal force distribution. The location, sequence, and failure criterion of the vulnerable failure region are also determined. Finally, a reinforcement method is proposed, based on the point of failure area, to ensure stable control of the ASUL on the surface.

This paper establishes a load-bearing mechanical model, studies the distribution law of the internal force of the bearing structure, and reveals the impact of the overlying load, horizontal pressure coefficient, and rise-to-span ratio on the bearing performance based on the morphological characteristics of the ASUL. The study facilitates the determination of the stability criterion for the ASUL. The reported results can provide a basis for applying arch structures to rock formation control in mining areas with thick unconsolidated layers.

### Arch structure in unconsolidated layers

**Figure 2:**
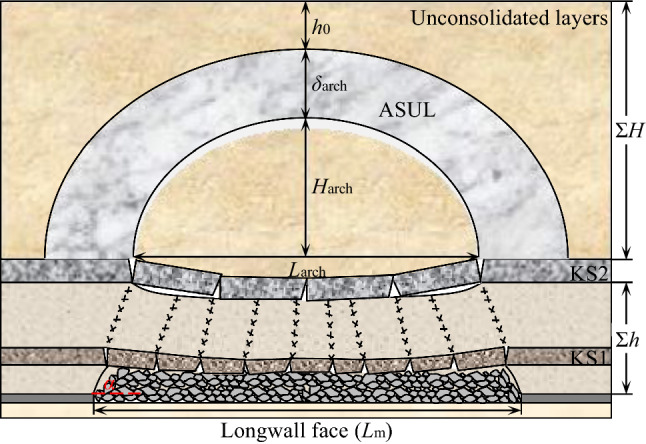
Diagram of the ASUL^22^.

Based on the diagram of the ASUL (Fig. 2), equations for the morphological characteristics, rise-to-span ratio, and thickness of the arch structure were derived through theoretical research^7,9,11,22^. The equation for calculating the critical thickness of the unconsolidated layers for forming an arch structure was obtained as shown in Eq. (1).

1 $$\left\{ {\begin{array}{*{20}l} {x^{2} - L_{{{\text{arch}}}} x + \lambda y^{2} + \frac{1}{{4H_{{{\text{arch}}}} }}\left( {L_{{{\text{arch}}}}^{2} - 4\lambda H_{{{\text{arch}}}}^{2} } \right)y = 0} \hfill \\ {\frac{{H_{{{\text{arch}}}} }}{{L_{{{\text{arch}}}} }} \ge \frac{{\sqrt {\left( {C + \gamma h_{0} \tan \varphi } \right)^{2} + \lambda \gamma^{2} h_{0}^{2} } { - }\gamma h_{0} \tan \varphi { - }C}}{{2\lambda \gamma h_{0} }}} \hfill \\ {\delta_{{{\text{arch}}}} = \frac{{\gamma h_{0} L_{{{\text{arch}}}} }}{{\left[ {\gamma \left( {h_{0} { + }iL_{{{\text{arch}}}} } \right)\tan (45^{ \circ } + \frac{\varphi }{2}) + 2C} \right]\tan (45^{ \circ } + \frac{\varphi }{2})\cos \varphi }}} \hfill \\ {H_{{\text{C}}} \ge \frac{{H_{{{\text{arch}}}} }}{{L_{{{\text{arch}}}} }}\left( {L_{m} { - }\frac{2\sum h }{{\tan \alpha }}} \right){\mkern 1mu} + \frac{{\gamma h_{0} H_{{{\text{arch}}}} }}{{\left[ {\gamma \left( {h_{0} { + }H_{{{\text{arch}}}} } \right)\tan (45^{ \circ } + \frac{\varphi }{2}) + 2C} \right]\tan (45^{ \circ } + \frac{\varphi }{2})\cos \varphi }}} \hfill \\ \end{array} } \right.$$

where *L*_arch_, *H*_arch_, *δ*_arch_, *H*_*C*_ (m) refer to the span, rise, thickness, and critical thickness of the unconsolidated layers, respectively; *γ* (kN/m^3^) refers to the bulk density of the rock strata; *α* (°) refers to the fracture angle; *C* (MPa) refers to the cohesion strength of the unconsolidated layers; *φ* (°) refers to the internal friction angle of the unconsolidated layers; *λ* refers to horizontal pressure coefficient;* L*_m_ (m) refers to the width of the working face; Σ*h* (m) refers to the distance between the bottom interface of the main critical layer and dome interface of the coal seam; *h*_0_ (m) refers to the thickness of the unconsolidated layers above the ASUL.

### Bearing performance of arch structure in unconsolidated layers

#### Mechanical model of the ASUL

According to the mechanics of granular media formed by the ASUL^23^, the structure simultaneously has axial forces, bending moments, and shear forces acting at the dome and base of the arch, which can be simplified into a hingeless arch structure statically indeterminate to the third degree in structural mechanics^24^. Due to the complexity of statically indeterminate structure analysis, the morphological characteristics of the hingeless arch are considered the same as those of a corresponding three-hinged arch. Thus, the three-hinged arch mechanical model was used to research the equations related to the morphological characteristics, rise-to-span ratio, and ASUL thickness^11^. However, when the bearing characteristics and internal force distribution law of the ASUL need to be analysed, a hingeless arch mechanical model is required.

**Figure 3:**
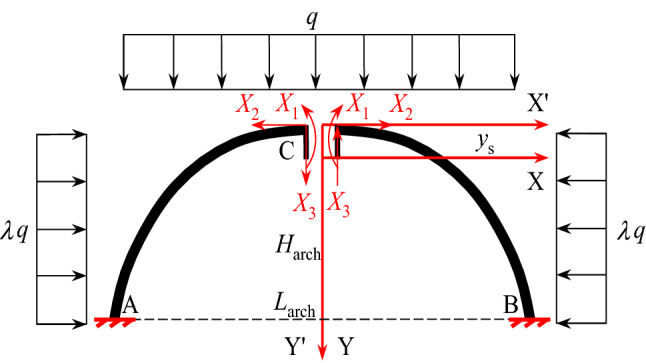
Mechanical model of the ASUL.

When the mechanical model is constructed, the unconsolidated layers are assumed to be uniformly distributed, and the ASUL thickness remains constant from the dome to the base of the arch. The mid-plane curve with load bearing in the ASUL is the primary focus of this study. Additionally, the overlying load on the ASUL is uniformly distributed and equal to the weight of the overlying unconsolidated layers, while the horizontal load is uniformly distributed and the horizontal pressure coefficient is constant. The mechanical model is shown in Fig. 3, containing fixed constraints at arch bases A and B. *L*_arch_, *H*_arch_, *q*, *λq*, and *λ* correspond to the span, rise, overlying load, horizontal load, and horizontal pressure coefficient of the ASUL, respectively.

#### Analytical solutions for internal force of the ASUL

As the hingeless arch is statically indeterminate to the third degree problem, analysis was conducted using the force method. Three unknown forces—*X*_1_, *X*_2_ and *X*_3_—were considered at the dome of the ASUL, corresponding the bending moment, axial force, and shear force, respectively. Their directions are shown in Fig. 3. According to material mechanics^25^, when a symmetrical structure is subjected to a symmetrical load, the anti-symmetrical internal force in the symmetrical cross-section is zero. Considering ASUL and load are both symmetrical structures, the anti-symmetrical internal force of the ASUL on the symmetrical cross-section of the arch dome is zero, i.e., *X*_3_ = 0. Based on the configuration equation of the ASUL, Eq. (1), the distance from the elastic centre of the ASUL to the arch dome, *y*_s_, is calculated as follows:

2 $$y_{s} = \frac{{\int {y\frac{ds}{{EI}}} }}{{\int {\frac{ds}{{EI}}} }} = \frac{b}{{4aL_{arch} }}\left( {L_{arch} \sqrt {4a^{2} - L_{arch}^{2} } + 4a^{2} \arcsin \frac{{L_{arch} }}{2a}} \right)$$

According to the basic structure diagram of the elastic centre method, the standard force method equation is shown in Eq. (3):

3 $$\left\{ {\begin{array}{*{20}l} {\delta_{11} X_{1} + \Delta_{{1{\text{p}}}} = 0} \hfill \\ {\delta_{22} X_{2} + \Delta_{{2{\text{p}}}} = 0} \hfill \\ \end{array} } \right.$$

where *δ*_11_ and *δ*_22_ denote the displacements due to the unit forces.*X*_1_ = 1 and *X*_2_ = 1, and *Δ*_1p_ and *Δ*_2p_ denote the displacements of point *C* along *X*_1_ and *X*_2_ under the action of the external forces, respectively.

The unknown forces, *X*_1_ and *X*_2_, shown in Fig. 3, are derived using Eq. (3):

4 $$\left\{ {\begin{array}{*{20}l} {X_{1} = \frac{{\left( {12H_{arch}^{2} \lambda + 5L_{arch}^{2} } \right)q}}{120}} \hfill \\ {X_{2} = \frac{{\left( {24H_{arch}^{2} \lambda + 7L_{arch}^{2} } \right)q}}{{56H_{arch} }}} \hfill \\ \end{array} } \right.$$

According to the equilibrium conditions of the ASUL in Fig. 3, the internal forces borne by any cross-section of the ASUL are as follows:

5 $$\left\{ {\begin{array}{*{20}l} {N(x) = X_{2} \cos \varphi + N_{P} } \hfill \\ {M(x) = X_{1} + X_{2} (y - y_{s} ) + M_{P} } \hfill \\ {Q(x) = X_{2} \sin \varphi + Q_{P} } \hfill \\ \end{array} } \right.$$

where *N(x)*, *M(x)*, and *Q(x)* are the axial force, bending moment, and shear force of the ASUL, respectively, and *N*_*P*_, *M*_*P*_, and *Q*_*P*_ are the axial force, bending moment, and shear force of the cross-section of the ASUL when bearing a load, respectively.

The axial force, bending moment, and shear force in the cross-section of the ASUL under overlying load *q* and horizontal load *λq* are as follows:

6 $$\left\{ {\begin{array}{*{20}l} {N_{P} = qx\sin \varphi - \lambda qy\cos \varphi } \hfill \\ {M_{P} = - \frac{q}{{2L_{{{\text{arch}}}}^{4} }}\left( {L_{{{\text{arch}}}}^{4} x^{2} + 16\lambda H_{{{\text{arch}}}}^{2} x^{4} } \right)} \hfill \\ {Q_{P} = - qx\cos \varphi - \lambda qy\sin \varphi } \hfill \\ \end{array} } \right.$$

Substituting Eq. (1) into Eq. (6), the analytical equations for calculating the axial force, bending moment, and shear force in the cross-section of the ASUL are as follows:

7 $$\left\{ {\begin{array}{*{20}l} {N\left( x \right) = \frac{q}{{56H_{arch} \sqrt {L_{arch}^{4} + 64H_{arch}^{2} x^{2} } }}\left\{ {\left[ {24L_{arch}^{2} \lambda - 224x^{2} \left( {\lambda - 2} \right)} \right]H_{arch}^{2} + 7L_{arch}^{4} } \right\}} \hfill \\ {M\left( x \right) = - \frac{{\lambda H_{arch}^{2} q}}{{70L_{arch}^{4} }}\left( {560x^{4} - 120L_{arch}^{2} x^{2} + 3L_{arch}^{4} } \right)} \hfill \\ {Q\left( x \right) = \frac{{8\lambda H_{arch}^{2} q}}{{7L_{arch}^{2} \sqrt {L_{arch}^{4} + 64H_{arch}^{2} x^{2} } }}\left( {3L_{arch}^{2} x - 28x^{3} } \right)} \hfill \\ \end{array} } \right.$$

#### Distribution characteristics for the internal forces of the ASUL

**Figure 4:**
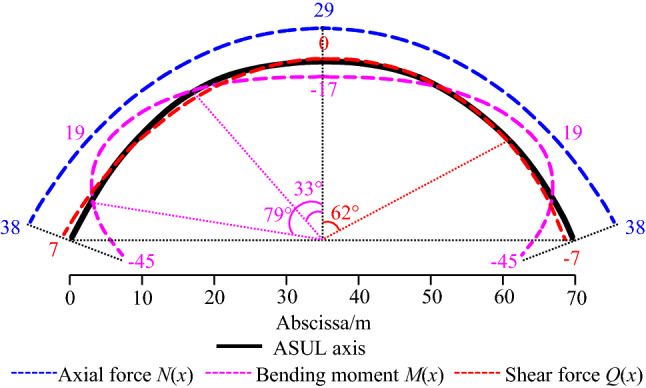
Internal force distribution of the ASUL (unit of force: kN, unit of bending moment: kN·m).

Figure 4 shows the internal force distribution law of the cross-section of the ASUL after substituting *H*_arch_ = 27 m, *L*_arch_ = 70 m, *q* = 1 kN/m, and *λ* = 0.54 into Eq. (7). When the internal force is less than zero, the direction of the internal force is opposite to that shown in the model in Fig. 3. The distribution for the load-bearing internal forces of the ASUL are characterised as follows:

1. Under overlying and horizontal loads, the axial force and bending moment in the cross-section of the ASUL are symmetrically distributed, and the shear force is anti-symmetrically distributed. The axial force is significantly greater than the shear force. The axial force is greater than the bending moment at all points of the ASUL except the base. The axial force increases from 29 kN at the dome of the arch to 38 kN at the base of the arch. The axial force is a sum of compressive stresses along the axis of the ASUL at all points.
2. The bending moment of the cross-section of the ASUL is divided into three areas along the axis direction. The bending moment is less than zero when both sides of the ASUL are in the range of 0°–33° and 79°–90°. Accordingly, the inner side of the arch body is strained while the outer side is compressed. The bending moment is greater than zero when both sides are in the range of 33°–79°. Accordingly, the inner side of the arch body is compressed, and the outer side is strained.
3. The maximum value of the shear force is only 15% of the axial force and 16% of the bending moment in the cross-section of the ASUL. Therefore, the impact of the shear force on the ASUL is not significant. The shear force is greater than zero at the dome and spandrel of the arch within 62° on both sides of the ASUL. Hence, due to the shear force, the dome and spandrel of the arch are prone to shear failure.

According to the internal force distribution law, under a load-bearing force, the ASUL primarily transmits the axial forces and load of the overlying unconsolidated layers through axial compression of the cross-section. Additionally, the axial pressure direction is consistent with the axial equation of the ASUL. When the compressive properties of the unconsolidated layers are significantly greater than its tensile and shear properties^8^, the ASUL serves as a stable bearing under axial forces. Due to the combination of the axial force and bending moment, the stability of the dome, spandrel, and base of the arch directly affect the bearing stability of the ASUL.

### Impact analysis of the bearing performance of arch structure in unconsolidated layers

**Figure 5:**
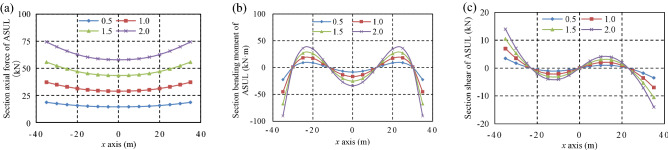
Internal force distribution of the ASUL under different loading: (**a**) axial force, (**b**) bending moment, (**c**) shear force.

**Figure 6:**
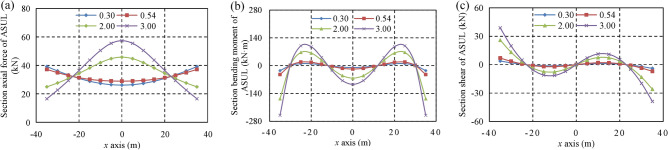
Internal force distribution of the ASUL under different horizontal pressure coefficients: (**a**) axial force, (**b**) bending moment, (**c**) shear force.

**Figure 7:**
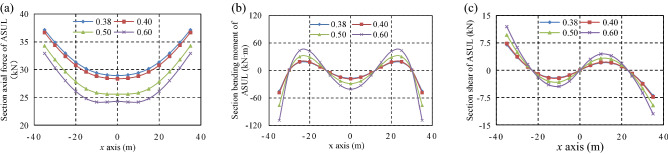
Internal force distribution of the ASUL under different rise-to-span ratio: (**a**) axial force, (**b**) bending moment, (**c**) shear force.

The overlying loads were set as 0.5 kN/m, 1.0 kN/m, 1.5 kN/m, and 2.0 kN/m to analyse the influence of the overlying load, horizontal pressure coefficient, and rise-to-span ratio on the load-bearing internal force distribution of the ASUL. Accordingly, the horizontal pressure coefficients are set as 0.30, 0.54, 2.00, and 3.00, respectively. Further, the rise-to-span ratios are set as 0.38, 0.40, 0.50, and 0.60, respectively. The increase in *H*_arch_ and *L*_arch_ was 27 m and 70 m, respectively. The internal force distributions of the ASUL under different loading, horizontal pressure coefficient, and rise-to-span ratio are shown in Figs. 5, 6 and 7, respectively. When the internal force is less than zero, the direction of the internal force is opposite to that set by the model in Fig. 3. When the bending moment is less than zero, the inner side of the ASUL is strained, and the outer side is compressed.

#### Overlying load of the ASUL

When the horizontal pressure coefficient and rise-to-span ratio are 0.54 and 0.38, respectively, the internal force in the cross-section of the ASUL under different overlying loads is shown in Fig. 5. The axial force and bending moment are symmetrically distributed while the shear force is anti-symmetrically distributed.

1. The axial force gradually increases from the dome to the base of the arch as the overlying load increases. The axial force at the dome and base of the arch increases from 14.5 to 57.9 kN and from 18.6 to 74.4 kN, respectively. Thus, the axial force at the base has the greatest increase compared to the spandrel and dome of the arch.
2. The bending moment is the smallest at the dome of the arch, followed by the spandrel, and the base, wherein it was the largest; bending moment increases as the overlying load gradually increases. The bending moment at the dome, spandrel, and base of the arch increases from 8.4 to 33.7 kN·m, from 9.0 to 36.0 kN·m, and from 22.5 to 90.0 kN·m, respectively. Thus, the bending moment at the base has the greatest increase compared to the dome and spandrel of the arch.
3. The shear force is the greatest at the base of the arch, followed by the arch spandrel; it is zero at the dome of the arch. The shear force at the base and spandrel of the arch gradually increases as the overlying load increases. The shear force at the spandrel and base of the arch increases from 2.0 to 4.0 kN and from 3.5 to 14.0 kN, respectively. Accordingly, the increase of shear force at the base is greater than that at the spandrel of the arch.
4. As the overlying load gradually increases, the shear force increases at the base and spandrel of the arch due to a significant overall increase in the axial force and bending moment at the base, spandrel, and dome of the arch, with the greatest increase at the base. Thus, the stability of the base, spandrel, and dome of the arch directly affects the overall stability of the ASUL.

#### Horizontal pressure coefficient of the ASUL

The internal forces in the cross-section of the ASUL under different horizontal pressure coefficients for an overlying load of 1.0 kN/m and rise-to-span ratio of 0.38 are shown in Fig. 6. The axial force and bending moment are symmetrically distributed, while the shear force is anti-symmetrically distributed. When the horizontal pressure coefficient is less than one, the overlying load on the ASUL is greater than the horizontal load. When the horizontal pressure coefficient is greater than one, the overlying load is less than the horizontal load.

1. When the horizontal pressure coefficient is < 1 and increases from 0.30 to 0.54, the horizontal load is lesser than the overlying load. Further, the change in the axial force, bending moment, and shear force is smaller. The axial force is greater than the bending moment, which is greater than the shear force. Additionally, the axial force gradually increases from the dome to the base of the arch. The bending moment is the largest at the base, followed by the spandrel, and smallest at the dome. Accordingly, the shear force is the largest at the base, followed by the spandrel, and zero at the dome.
2. When the horizontal pressure coefficient is > 1 and increases from 2.00 to 3.00, the horizontal load is greater than the overlying load. ASUL is primarily affected by the horizontal load and bending moment. The bending moment is greater than the axial force, which is greater than the shear force. The axial force decreases gradually from the dome to the base of the arch due to the horizontal load when the horizontal pressure coefficient is less than one.
3. As the horizontal pressure coefficient increases, the bending moment significantly increases at the dome, spandrel, and base of the arch. The bending moment at the dome, spandrel, and base of the arch increases from 64 to 94 kN·m, 67 to 100 kN·m, and 167 to 250 kN·m, respectively. Accordingly, the increase in the bending moment is the maximum at the base of the arch. Additionally, as the horizontal pressure coefficient increases, the shear force at the spandrel and base of the arch increases from 8 to 11 kN and from 26 to 39 kN, respectively.

#### Rise-to-span ratio of the ASUL

The internal forces in the cross-section of the ASUL under different rise-to-span ratios for an overlying load of 1.0 kN/m and horizontal pressure coefficient of 0.54 are shown in Fig. 7. The axial force and bending moment are symmetrically distributed, and the shear force is anti-symmetrically distributed.

1. The axial force gradually decreases from the dome to the base of the arch as the rise-to-span ratio gradually increases. The axial force at the dome and base of the arch decreases from 29 to 24.3 kN and 37.2 to 33 kN, respectively. Accordingly, the axial force at the base decreases the least when compared to the spandrel and dome of the arch.
2. The bending moment is the smallest at the dome of the arch, followed by the spandrel, and largest at the base. It increases at the dome, spandrel, and base of the arch as the rise-to-span ratio gradually increases. The bending moment at the dome, spandrel, and base of the arch increases from 16.9 to 40.8 kN·m, 18.0 to 43.4 kN·m, and 45.0 to 108.9 kN·m, respectively. Accordingly, the bending moment at the base increases the most when compared to the dome and spandrel of the arch.
3. The shear force is the largest at the base of the arch, followed by the spandrel, and zero at the dome. The shear force at the base and spandrel of the ASUL gradually increases with the rise-to-span ratio. The shear force at the spandrel and base of the arch increases from 2.0 to 4.4 kN and 7 to 12.0 kN, respectively. The shear force has the greatest increase at the base of the arch.
4. As the rise-to-span ratio gradually increases, the ASUL gradually transforms from a horizontal flat to a vertical flat shape. The shear force at the base and spandrel of the arch increases due to a significant increase in the axial force and bending moment at the base, spandrel, and dome of the arch. The shear force at the base has a more significant increase than at the spandrel and dome of the arch.

### Failure criterion of the arch structure in unconsolidated layers

#### Analysis of the bearing failure processes of the ASUL

Under overlying load, the cross-section stresses of the load-bearing ASUL consist of the axial force, bending moment, and shear force. The axial force gradually increases from the dome to the base of the arch and acts parallel to the arch axis. A stable load-bearing ASUL is achieved through axial compressive stress. Compared to the axial force and bending moment, the shear force has a relatively small impact on the stability of the structure.

Under actual mining conditions, failure of the ASUL must have a point of origin. The damage spreads to other locations of the arch body in succession due to a chain effect, eventually causing subsequent failure of the ASUL. During the mining process of the working face, the base of the arch acts on the upper part of the rock strata on both sides of the working face, as shown in Fig. 2. According to Eq. (1), when the width, *L*_m_, of the working face is constant, the rise and span of the ASUL is constant and the base maintains stability. When the width of the working face increases, the rock strata on both sides of the working face ruptures, and the base fails consequently. Therefore, the failure and forward movement of the base of the ASUL is due to an increase in the cut width of the working face.

**Figure 8:**
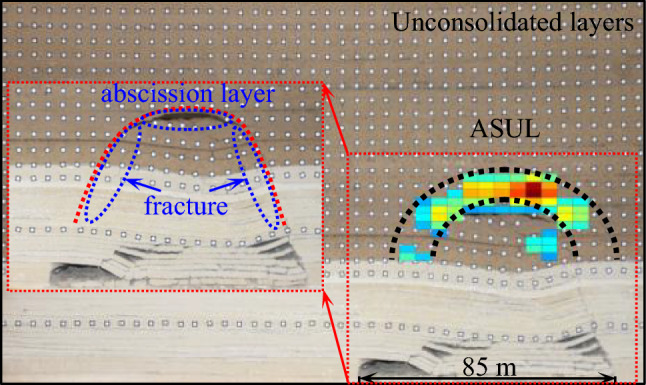
Morphological characteristics of the ASUL.

According to the simulation results of the spatial morphological evolution law of the ASUL^11^, a separate cracked area is formed between the inner side of the dome and the spandrel of the ASUL, as shown in Fig. 8. Due to the cracked area, the support offered by the ASUL to the collapsed unconsolidated layers disappears and decreases at the dome and spandrel of the arch, respectively. According to the results of bearing performance analysis, the ASUL is susceptible to damage at the dome and spandrel of the arch under load.

Therefore, when mining the working face, the failure chronology of the ASUL is as follows: an increase in the cut width of the working face causes failure and forward movement of the base of the ASUL, resulting in compression failure of the dome and spandrel of the arch, further inducing a lift at the dome of the arch, causing subsequent failure of the ASUL. Consequently, a new ASUL is formed.

#### Failure criterion of the ASUL.

1. Compression failure of the ASUL

As shown in Fig. 4, the axial force of the cross-section at the dome of the ASUL is the compressive stress. The bending moment is less than zero, indicating that the bending moment is in the clockwise direction. Accordingly, the inner side of the dome of the arch is under tensile stress while the outer side is under compressive stress, and the shear force of the cross-section at the dome of the arch is zero. Assuming the width of the cross-section of the ASUL to be *b*_arch_, *x* = 0 at the dome of the arch, and *y* = *H*_arch_, according to Eq. (7). The compressive and tensile stresses at the dome of the arch due to the axial force and bending moment, respectively, are calculated using Eq. (8):

8 $$\left\{ {\begin{array}{*{20}l} {\sigma_{N} \left| {_{{{\text{dome}}}} } \right. = \frac{{q(24\lambda H_{arch}^{2} + 7L_{arch}^{2} )}}{{56H_{arch} b_{arch} \delta_{arch} }}} \hfill \\ {\sigma_{M} \left| {_{{{\text{dome}}}} } \right. = - \frac{{18\lambda qH_{{_{arch} }}^{2} }}{{35b_{arch} \delta_{{_{arch} }}^{2} }}} \hfill \\ \end{array} } \right.$$

Therefore, the combined stress in the dome of the arch is:

9 $$\sigma_{c} \left| {_{{{\text{dome}}}} } \right. = \frac{{q(120\lambda \delta_{{_{arch} }}^{2} H_{{_{arch} }}^{2} + 35\delta_{arch} L_{{_{arch} }}^{2} - 144\lambda H_{{_{arch} }}^{3} )}}{{280b_{{_{arch} }}^{2} H_{arch} \delta_{{_{arch} }}^{2} }}$$

According to Eq. (9), *σ*_c_|_dome_ > 0, and the combined force at the dome of the ASUL is compressive stress. The type of failure of the dome of the arch is compression failure. When *σ*_c_|_dome_ > [*σ*_C_] ([*σ*_C_] is the maximum compressive strength of the unconsolidated layers), the dome undergoes compression failure.

(B) Compression failure of the spandrel of the ASUL

As shown in Fig. 4, the axial force at the spandrel of the ASUL is compressive stress, and the bending moment of the cross-section at the spandrel is greater than zero, indicating that the inner side of the spandrel of the arch is under compressive stress while the outer side is under tensile stress. The shear force has a smaller impact on the spandrel of the arch and is therefore negligible. At the spandrel, *x* = $\sqrt{\frac{3}{28}} L_{{{\text{arch}}}}$. According to Eq. (7), the compressive and tensile stresses of the cross-section of the spandrel of the ASUL due to the axial force and bending moment, respectively, are calculated using Eq. (10):

10 $$\left\{ {\begin{array}{*{20}l} {\sigma_{N} \left| {_{{{\text{spandrel}}}} } \right. = \frac{{qL_{arch} \sqrt {336\lambda H_{arch}^{2} + 49L_{arch}^{2} } }}{{56H_{arch} b_{arch} \delta_{arch} }}} \hfill \\ {\sigma_{M} \left| {_{{{\text{spandrel}}}} } \right. = \frac{{72\lambda qH_{{_{arch} }}^{2} }}{{245b_{arch} \delta_{{_{arch} }}^{2} }}} \hfill \\ \end{array} } \right.$$

Therefore, the combined stress at the spandrel of the ASUL is:

11 $$\sigma_{c} \left| {_{{{\text{spandrel}}}} } \right. = \frac{{q(576\lambda H_{arch}^{3} + 35\delta_{arch} L_{arch} \sqrt {49L_{arch}^{2} + 336H_{arch}^{2} } )}}{{1960b_{arch} H_{arch} \delta_{arch}^{2} }}$$

According to Eq. (11), *σ*_c_|_spandrel_ > 0, the combined force at the spandrel of the ASUL is compressive stress, and the type of failure at the spandrel of the arch is compression failure. When *σ*_c_|_spandrel_ > [*σ*_C_], the spandrel undergoes compression failure.

Equations (9) and (11) show that when the compressive stress on the dome and spandrel of the ASUL exceeds the maximum compressive strength of the unconsolidated layers, the structure undergoes failure. Failure occurs as compression failure at the dome and spandrel of the ASUL.

## Discussions

During the mining process of the working face, the ASUL controls the movement and deformation of the overlying strata and ground surface. During load-bearing, the body of the arch is affected by axial force, bending moment, and shear force. The axial force is greater than the bending moment, which is greater than the shear force, except at the base of the arch. The ASUL maintains its own stability and has a load-bearing role on the overlying strata and ground surface through axial compression. Under the overlying load, the base of the arch does not easily fail when acted upon the upper part of the stable rock strata when no cracks exist on either side of the working face. Upon impact from the collapsed unconsolidated layers, the lower part of the dome of the arch experiences bed separation, and the spandrel of the arch cracks, as shown in Fig. 8. The supporting effect of the collapsed unconsolidated layers on the ASUL disappeared at the dome and decreases at the spandrel. Furthermore, the ASUL is prone to failure at the dome and spandrel. Therefore, failure of the ASUL is summarized as follows: an increase in the cut width of the working face causes failure and a forward movement of the base of the ASUL, resulting in compression failure at the dome and spandrel of the arch, further inducing a lift in the dome of the arch, resulting in failure of the ASUL. Consequently, a new ASUL is formed.

**Figure 9:**
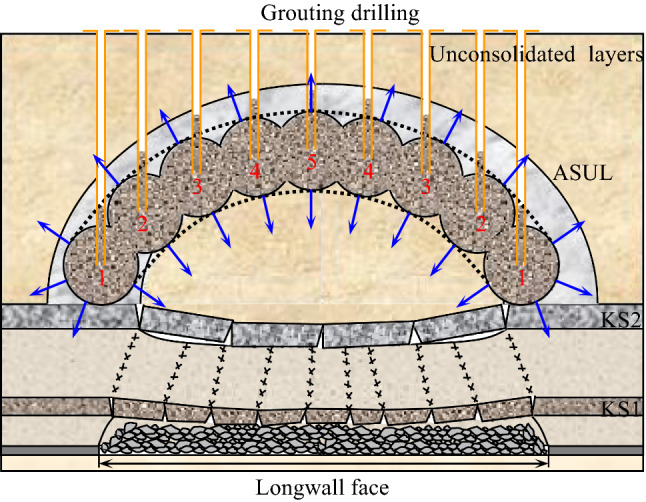
ASUL reinforcement through compaction grouting.

In mining regions with thick unconsolidated layer distribution, when the thickness of the ASUL meets the critical thickness, as shown in Eq. (1), an ASUL can be formed. The stability of the ASUL directly affects the stability of the overlying strata and ground surface. Previous studies have demonstrated that the characteristics of the overlying strata movement and surface subsidence pattern of coal mining in the mining regions with thick unconsolidated layers are characterised by sensitivity, severe subsidence, high subsidence coefficient, and high subsidence rate^15,26^, resulting in large-scale surface subsidence^7,10,27,28^. Based on the results of our research on the bearing performance of the ASUL, a control method that reinforces the surface subsidence of the ASUL by compaction grouting is proposed, as shown in Fig. 9, using the following processes:

1. Before mining the working face that involves thick unconsolidated layers, test experiments should be conducted to determine the basic physical and mechanical parameters of the unconsolidated layers and rock strata by drilling and sampling based on the geological mining conditions of the working face and drillhole column.
2. The working face and mechanical parameters of the unconsolidated layers and rock strata are substituted into Eq. (1) to determine the span, rise, and thickness of the ASUL. Further, the stability of the ASUL is discerned using Eqs. (9) and (11).
3. When the ASUL can bear load stably, the working face is mined normally. When the ASUL undergoes failure, it must be reinforced via compaction grouting.
4. A high-pressure grouting pump is used to drill holes through the ground at specific locations in the unconsolidated layers. The final hole location of the grouting drillhole is determined according to the rise of the ASUL, calculated using Eq. (1). The grouting pressure is determined according to the original rock stress at the final hole location of the grouting drillhole. Coal ash is used as the primary grouting material, and a certain proportion of cement is added to the slurry to increase the bonding activity of the coal ash slurry. The distance between the drillholes is determined based on the slurry diffusion radius. Based on our previous studies and engineering practice results, the effective diffusion radius of this slurry is between 10 and 20 m.
5. Using the 'one-time, upward, staged, and multiple-ground-drilling' process, grouting is undertaken in the order of 1, 2, 3, 4, and 5. After grouting, the structural body formed by the solidification of the slurry and unconsolidated layers is arched and beaded from the bottom to the top. During grouting, the arched and beaded structure can modify and consolidate the unconsolidated layers within the range of the slurry diffusion radius to strengthen them. Conversely, the grouting pressure can compress and consolidate adjacent unconsolidated layers. During extraction on the working face, the arch bead-like structure, combined with the ASUL, serves as a bearing control for the overlying strata and ground surface, further decreasing the deformation of the ASUL, overlying strata, and ground surface. The combined structure also controls the ground subsidence induced by coal mining in mining regions with thick unconsolidated layers.

This method has the following limitations. When the thickness of the unconsolidated layers is small, the method is only applicable the first working face. When the thickness of the unconsolidated layers is large, the method is not only applicable to the first working face, but also to the other working faces. When ASUL of the first working face is reinforced, the mining of the second working face will affect the first working face, so ASUL reinforcement should cover all working faces in the whole area. The diffusion radius of grouting drilling slurry needs to be determined by field measurement.

ASUL reinforcement through compaction grouting was carried out before mining in 3–1 working face of a mine in Shandong Province. The dip angle of coal seam is 4–14°, and the thickness of coal seam is 4.0–5.8 m. The average thickness of the overlying unconsolidated layer is 295 m, and the average mining depth is 547 m. The advancing length of the working face is 612 m, and the width of the working face is 176 m. The measured diffusion radius of grouting drill in 3–1 working face is 17 m, and the rise-to-span ratio of ASUL is 0.69 by calculation. A total of 21 grouting holes were arranged in the 3–1 working face. The maximum surface subsidence value of 3–1 working face is 392 mm, and the surface subsidence in the whole area does not exceed 500 mm. Through this method, 600,000 tons of coal resources were recovered, and the effect of control surface subsidence is significant.

## Conclusions

1. A mechanical model for load bearing of the ASUL was established to determine an analytical expression for the axial force, bending moment, and shearing force of the cross-section. Under overlying and horizontal loads, the axial force and bending moment in the cross-section of the ASUL were symmetrically distributed, while the shear force was anti-symmetrically distributed. The axial force gradually increased from the dome to the base of the arch and experienced compressive stress along the axis in all regions of the ASUL. The bending moment was the highest at the base, followed by the spandrel, and the least at the dome of the arch. Due to the bending moment, the inner side of the dome of the ASUL was strained, and the outer side was compressed. Compared to the axial force and bending moment in the cross-section of the ASUL, the shear force had a smaller impact on its stability. The ASUL transmitted the axial force and load of the overlying unconsolidated layers through axial compression of the cross-section, maintained its stability, and played a load-bearing role for overlying unconsolidated layers.
2. The distribution law of the overlying load, horizontal pressure coefficient, and rise-to-span ratio of the internal force for the ASUL was determined. As the overlying load and rise-to-span ratio of the ASUL increased, the axial force, bending moment, and shear force of the cross-section of the ASUL gradually increased; they also increased at the dome, spandrel, and base of the arch, respectively. Compared to the dome and base, the axial force, bending moment, and shear force at the base of the arch had the greatest increase. When the horizontal pressure coefficient was < 1, the axial force gradually increased from the dome to the base of the arch, and when it was > 1, the axial force gradually decreased. As the horizontal pressure coefficient increased, the bending moment, shear force, and axial force at the dome of the arch gradually increased, while the axial force at the base of the arch gradually decreased. Similarly, the axial force, bending moment, and shear force had the greatest increase at the base of the arch. Thus, the stability of the dome, spandrel, and base of the arch directly impact the stability of the ASUL under load.
3. The failure criterion of load bearing of the ASUL was determined. Due to the combined effect of the axial force and bending moment of the cross-section of the ASUL, the top and spandrel of the ASUL were prone to compression failure. Based on this observation, the stability criterion for load-bearing of the ASUL corresponding to the compression failure of the dome and spandrel was obtained. When mining the working face, the ASUL served as a bearing control on the overlying unconsolidated layers. Furthermore, an increase in the cut width of the working face resulted in failure and forward movement of the base of the ASUL, resulting in compression failure of the dome and spandrel of the arch and inducing a lift of the dome of the arch, subsequently causing failure of the ASUL.
4. Method that reinforces the surface subsidence of the ASUL by compaction grouting was proposed. After implementing surface drilling and the 'one-time, upward, staged, and multiple-ground-drilling' process for compaction grouting, the structural body formed by the solidification of the slurry and unconsolidated layers was arched and beaded from the bottom to the top. During extraction on the working face, the arch bead-like structure, combined with the ASUL, served as a bearing control on the overlying strata and ground surface, reducing the deformation of the ASUL, overlying strata, and ground surface. It also controlled the ground subsidence induced by coal mining in mining regions with thick unconsolidated layers. However, further research on the control of surface subsidence by compaction grouting for reinforcing the ASUL is required, including the mechanism for surface subsidence control, proportion of grouting materials, and grouting process for different characteristics of unconsolidated layers.

## Data Availability

The datasets used and/or analyzed during the current study are available from the corresponding author on reasonable request.
